# DNA Methylation Patterns in Rat Mammary Carcinomas Induced by Pre- and Post-Pubertal Irradiation

**DOI:** 10.1371/journal.pone.0164194

**Published:** 2016-10-06

**Authors:** Masaru Takabatake, Benjamin J. Blyth, Kazuhiro Daino, Tatsuhiko Imaoka, Mayumi Nishimura, Masahiro Fukushi, Yoshiya Shimada

**Affiliations:** 1 Department of Radiation Effects Research, National Institute of Radiological Sciences, National Institutes for Quantum and Radiological Science and Technology, Chiba, Japan; 2 Department of Radiological Sciences, Graduate School of Human Health Sciences, Tokyo Metropolitan University, Tokyo, Japan; University of North Carolina at Chapel Hill School of Medicine, UNITED STATES

## Abstract

Several lines of evidence indicate one’s age at exposure to radiation strongly modifies the risk of radiation-induced breast cancer. We previously reported that rat mammary carcinomas induced by pre- and post-pubertal irradiation have distinct gene expression patterns, but the changes underlying these differences have not yet been characterized. The aim of this investigation was to see if differences in CpG DNA methylation were responsible for the differences in gene expression between age at exposure groups observed in our previous study. DNA was obtained from the mammary carcinomas arising in female Sprague-Dawley rats that were either untreated or irradiated (γ-rays, 2 Gy) during the pre- or post-pubertal period (3 or 7 weeks old). The DNA methylation was analyzed using CpG island microarrays and the results compared to the gene expression data from the original study. Global DNA hypomethylation in tumors was accompanied by gene-specific hypermethylation, and occasionally, by unique tumor-specific patterns. We identified methylation-regulated gene expression candidates that distinguished the pre- and post-pubertal irradiation tumors, but these represented only 2 percent of the differentially expressed genes, suggesting that methylation is not a major or primary mechanism underlying the phenotypes. Functional analysis revealed that the candidate methylation-regulated genes were enriched for stem cell differentiation roles, which may be important in mammary cancer development and worth further investigation. However, the heterogeneity of human breast cancer means that the interpretation of molecular and phenotypic differences should be cautious, and take into account the co-variates such as hormone receptor status and cell-of-origin that may influence the associations.

## Introduction

Exposure to radiation, either accidentally or for medical reasons, is associated with an increased incidence of breast cancer [1, 2] and several lines of evidence have indicated that one’s age at exposure to radiation, particularly at young ages, strongly modifies the risk of breast cancer [3, 4]. An integrated computational-experimental study has shown that stem cells in the mammary gland increase self-renewal and de-differentiation after irradiation in the juvenile stage, while adult stem cells do not show increased the self-renewal [5]. However, understanding the basis of differences in cancer susceptibility with age at exposure is challenging, with human cancer also heavily influenced by individual differences such as lifestyle (e.g., diet and parity) and genetic factors [6]; whereas in animal models, such factors can be controlled to provide an opportunity to study the effects of age in isolation [7–10]. Rat mammary cancer is a useful model of human breast cancer, mimicking the pathogenesis and hormone receptor expression of human breast cancer [11]. We previously reported that rat mammary carcinomas induced by pre- and post-pubertal irradiation have distinct gene expression patterns and a different balance of hormone receptor status [12]. Although there were changes in gene expression between normal mammary gland and radiation-induced tumors that were in common between the two age groups, there was a much larger set of genes which were either up- or down-regulated in tumors after post-pubertal irradiation that were unchanged from normal levels in tumors arising after pre-pubertal irradiation (i.e. the pre-pubertal tumors showed a more normal-like gene expression profile). The genes which were differentially expressed between the age groups were included in many functional categories within broad groups such as: tissue organization and development; cell fate; cell-cell communication; and, responses to signals such as steroid hormones and inflammation. The radiation-induced tumors from both ages showed a pattern of genomic aberrations, particularly deletions [13], that are characteristic of radiation-induced cancers [14–18], but no differences that might explain the gene expression differences.

Changes in gene expression in normal tissues are regulated at several levels such as through DNA methylation; histone modifications and other chromatin marks; and, the activity of transcription factors, repressors and other DNA-binding proteins [19]. DNA methylation profiles vary with developmental stage in normal tissues, including in mammary glands [20, 21], and underlie the differentiation of cell lineages within a given tissue [22]. DNA methylation also mediates the silencing of genes during breast cancer development [23–28], where global DNA hypomethylation is often seen alongside site-specific hypermethylation of CpG islands in the regulatory elements of tumor suppressor genes [29]. Differences in gene expression in the rat mammary cancers that depend on the age-at-exposure could be related to a different cell-of-origin, age-related differences in a common cell-of-origin, or divergence during tumor development despite a common starting point. Understanding whether the age-specific gene expression changes are associated with distinct DNA methylation profiles could help to explain the basis of the differences.

Here, our previously reported set of rat mammary carcinomas induced by pre- or post-pubertal irradiation were re-examined for changes in genome-wide DNA methylation patterns. The results indicate that although all of the radiation-induced tumors show dramatic changes in DNA methylation from the underlying normal mammary tissue, there are changes in CpG island DNA methylation characteristic of the age at radiation exposure which correspond to previously identified differences in gene expression. However, most of the gene expression differences identified were not associated with significant changes in DNA methylation at nearby CpG islands, implying other regulatory mechanisms are responsible for the majority of the age-specific gene expression signature.

## Materials and Methods

### Tissue Samples

All tissues were taken from a previously reported animal experiment [12]. Briefly, pre- and post-pubertal (3 and 7 weeks old, respectively) female Sprague-Dawley rats (Jcl:SD, Clea Japan, Tokyo, Japan) were subjected to single whole-body γ irradiation at a dose of 2 Gy from a ^137^Cs source (Gammacell 40; Nordion International, Ontario, Canada) or were not treated. All rats were fed a standard laboratory animal diet (CE-2; Clea Japan) until 9 weeks of age and then switched to AIN-76A diet containing 23.5% corn oil (Clea Japan) to potentiate mammary tumor development. Each rat was palpated weekly to detect mammary tumors. Spontaneous mammary carcinomas were obtained from the non-irradiated rats. Detailed information of the mammary tumors and normal mammary tissues is shown in S1 Table. All animal experiments were approved by the Institutional Animal Care and Use Committee of the National Institute of Radiological Sciences (Approval Numbers 14B-239 and 17–1012). The hormonal status of radiation-induced mammary carcinomas was described in our previous study. Briefly, mammary carcinomas were mostly estrogen receptor (ER) positive after post-pubertal exposure, but ER negative after pre-pubertal exposure, while tumors were positive and negative for progesterone receptor (PR) expression in both groups (S1 Table).

### Whole-genome methylation analysis of CpG Islands

The method and the CpG island DNA methylation microarray data described here have been deposited in the Gene Expression Omnibus (www.ncbi.nlm.nih.gov/geo) under accession number GSE62383. Briefly, genomic DNA was isolated from tumor tissues by proteinase K digestion followed by phenol/chloroform extraction. After sonication to produce DNA fragments of 200–600 bp, the MethylMiner Methylated DNA Enrichment Kit (Life Technologies, Carlsbad, CA) was used to enrich methylated DNA from one half of the DNA sample while the other half acted as the reference DNA. Enriched and reference DNA fragments were fluorescently labeled using the Agilent Genomic DNA Labeling Kit (Agilent Technologies, Santa Clara, CA), before purification and competitive hybridization using the Agilent DNA Hybridization Kit (Agilent) for 40 h to a 105K rat CpG island microarray (Agilent, Design ID: 021332) covering 96% of regions (approximately 8,000 genes) that are annotated as CpG islands in the rat genome (Nov. 2004, Baylor 3.4/rn4). The fluorescence data extracted from the image of the scanned slides was analyzed using the Agilent Genomic Workbench software (version 6.5).

### Data analysis and selection strategy

In the analysis algorithm, the degree of enrichment (or de-enrichment) by the methylation-enrichment step was compared to the overall distribution of enrichment for probes of the same melting temperature to calculate the probability that the CpG island represented by each probe was methylated or unmethylated. This was expressed ultimately as the log of the odds ratio (probability of methylated/probability of unmethylated, hereafter: LogOdds), such that a LogOdds score of 0 is equally likely to be methylated/unmethylated, while large absolute values are increasingly likely to be methylated or unmethylated (positive values or negative values, respectively). To identify significant differences in DNA methylation, independent samples T-tests for the mean of the LogOdds between the various groups were performed probe-wise, with a further minimum difference in the mean LogOdds between the groups applied (to exclude significant yet trivial differences). Where multiple probes were annotated to a single gene, only a single significant probe was necessary to include the gene as a candidate. The various *P*-value and LogOdds minimum difference thresholds used in the analyses are shown in the results. Using the previously published gene expression data [12], available for 4/7 pre-pubertal tumors, 6/7 post-pubertal tumors and 3/3 normal mammary glands (publicly available via GEO accession number GSE22770, www.ncbi.nlm.nih.gov/geo), the mean difference in relative gene expression between the groups was also applied to select candidates where CpG island DNA methylation differences corresponded to functional changes in gene expression (no bias was applied for any assumed direction of the correlation between methylation and gene expression). Univariate ANOVA was used to model the effect of age-at-exposure (pre-pubertal versus post-pubertal) and/or PR status (positive versus negative) on the LogOdds scores for probes which were initially identified as having age-dependence, in order to detect interactions or co-variate effects. Functional analysis of candidate gene lists was performed using the GeneMANIA online tool (available at: http://www.genemania.org/), using a false discovery rate threshold of *P* < 0.05.

### Validation experiments of bisulfite DNA sequencing

Genomic DNA was modified with sodium bisulfate using Chemicon’s CpGenome Fast DNA Modification Kit (Merck Millipore) according to the manufacturer’s instructions. Bisulfite-modified DNA was amplified by PCR using an Epitaq Kit (Takara Bio, Otsu, Japan). The genomic region and primers used for bisulfite DNA sequencing are shown in S2 Table. Amplified DNA products were cloned using an Invitrogen TOPO Cloning Kit (Life Technologies) and at least 10 individual clones containing the products from each primer set were sequenced, to calculate the average methylation level across all of the CpG sites.

## Results

### DNA Methylation Profiles of Rat Mammary Carcinomas and Normal Mammary Glands

**Fig 1:**
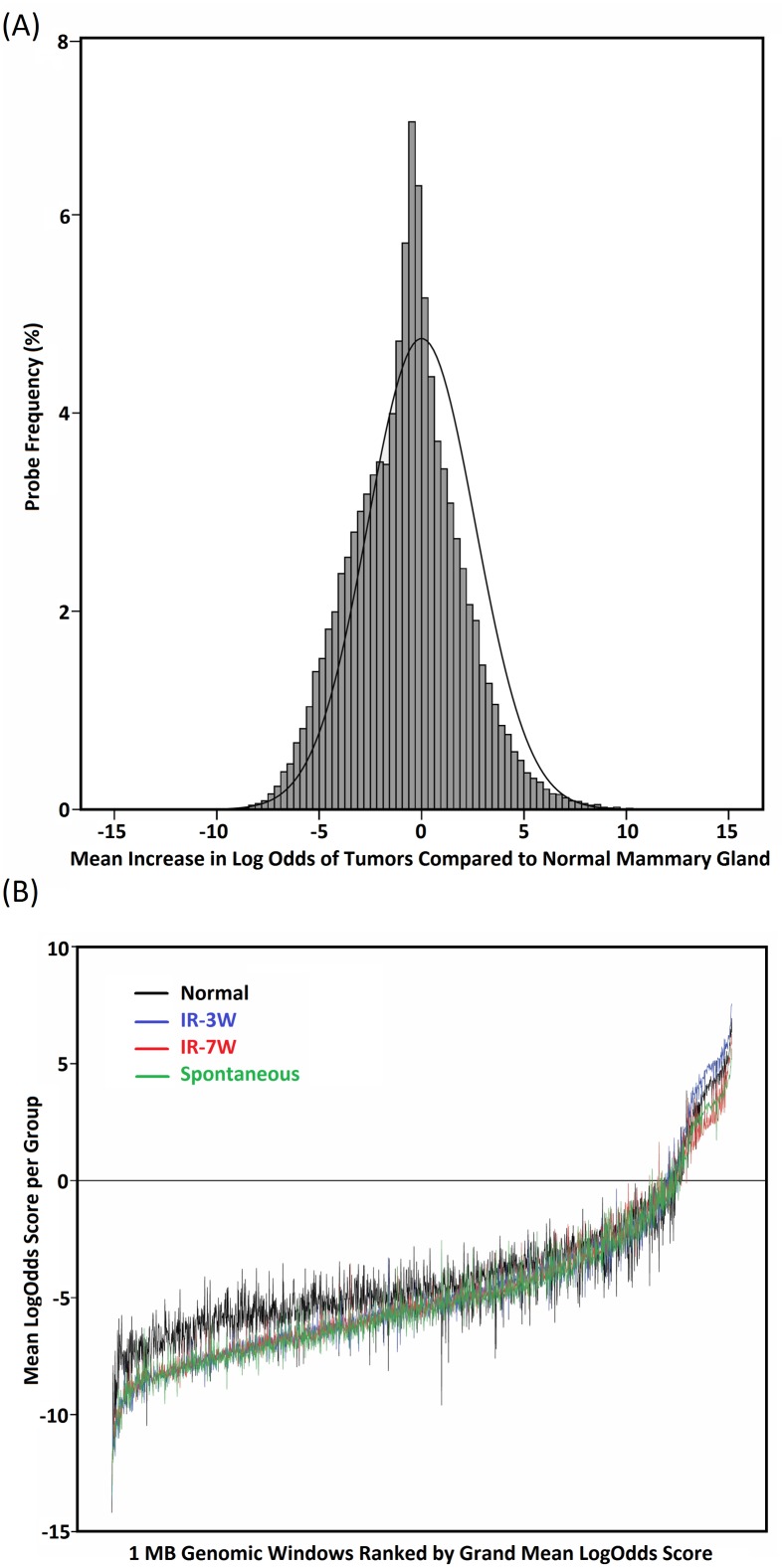
Distribution of differences of DNA methylation between normal mammary glands and mammary carcinomas. (A) Frequency histogram of the mean difference in LogOdds between the normal tissues (*n* = 3) and all of the mammary tumors (*n* = 21), where positive numbers indicate the frequency of probes with tumor hypermethylation, zero shows probes with no change, and negative numbers show tumor hypomethylation. The curve shows a simulated normal distribution (mean = 0, standard deviation = 2.5) with the same spread as the data, but centered on zero to indicate the null hypothesis of no global trend. The negative shift of the data shows the global trend towards tumor hypomethylation. (B) Changes in tumor DNA methylation levels by genomic regions. The mean LogOdds for the pre-pubertal (IR-3W, blue), post-pubertal (IR-7W, red) and spontaneous (green) tumors and for the normal mammary glands (black) are shown averaged across all probes within 1 MB intervals across the genome, with the genomic regions sorted by the grand mean LogOdds score (from less methylated genomic regions on the left through to the most densely methylated regions on the right). Tumor hypomethylation was not randomly distributed throughout the genome, with greater decreases in the tumors observed on the left in the less methylated genomic regions, with little to no difference in the regions with higher methylation levels in normal mammary glands.

Genome-wide DNA methylation analysis at CpG islands was carried out on rat mammary carcinomas from three groups (pre-pubertal irradiation, IR-3W, *n* = 7; post-pubertal irradiation, IR-7W, *n* = 7; spontaneous, *n* = 7) plus a small set of normal mammary gland tissues (*n* = 3) to indicate a baseline methylation level for the purposes of identifying global methylation trends in the tumors. Examination of DNA methylation in the whole set of 21 mammary tumors compared to normal mammary gland tissue revealed a general loss of methylation across the whole population of CpG island probes, consistent with the global hypomethylation frequently observed in cancer (Fig 1A) [30, 31]. However, the demethylation was not uniform across the genome, with genomic regions that showed the lowest DNA methylation levels in normal mammary tissue experiencing further loss of methylation (Fig 1B), while the half of 1 MB genomic regions with the highest baseline levels of methylation showed little change. The trend towards global hypomethylation in rat mammary carcinomas was also observed in LINE1 repeat elements (S1 Fig).

**Fig 2:**
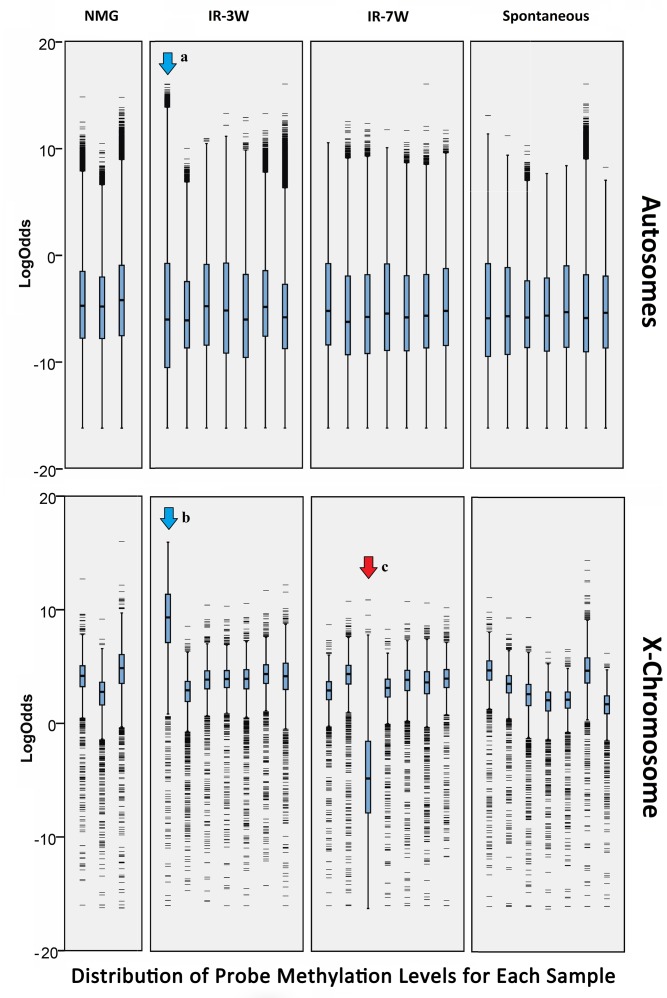
Global DNA methylation patterns unique to specific tumors. The distribution of LogOdds scores for all probes are shown as box plots for each sample, pooled for all the autosomes (top panel) and only the X chromosome (bottom panel). For autosomes, the DNA methylation scores have a very wide spread, where the rare heavily-methylated CpG islands can be seen as the outliers (thin black bars). In one pre-pubertal tumor (IR-3W, blue arrow, a), although the bulk of CpG island probes are similar to the other tumors, the population of heavily-methylated CpG islands show much higher methylation scores. For the X-chromosomes, the majority of all CpG island probes show a more uniform highly-methylated state, with less methylated probes only present as outliers. The same pre-pubertal tumor as before now shows the increased methylation of highly methylated regions affecting the bulk of the probes (blue arrow, b) suggesting aberrant regulation of methylation specifically affecting highly-methylated CpG islands. In contrast, one post-pubertal tumor (IR-7W, red arrow, c) shows a decrease in methylation affecting all of the probes on the X-chromosome, despite showing no change in the autosomes.

There were specific tumors which exhibited unique methylation patterns (Fig 2), with one tumor showing markedly increased DNA methylation on all chromosomes in genomic regions with high baseline methylation in normal mammary tissue, and another with dramatic demethylation only affecting the X chromosome. These might be explained by tumor-specific mutations in methylation control pathways and non-disjunction of activated/inactivated X chromosomes [32], respectively.

Since such a large proportion of CpG islands across the genome showed decreased methylation levels, and these were correlated with large-scale chromatin regions, it was difficult to select from the large set of genes with altered methylation levels a set of candidate genes which were specifically de-methylated in rat mammary carcinomas. Yet, despite the overwhelming trend towards loss of methylation, some CpG islands were observed to have significantly increased DNA methylation in tumors compared to normal mammary tissue (13.6% of those with significant differences, *P* < 0.001), and many of the genes associated with these CpG islands (selecting the quartile with the largest increases in LogOdds scores) were found to have >2-fold changes in gene expression levels (S2 Fig). These genes which went against the global trend are more likely candidates for cancer-specific epigenetic regulation of gene expression. The 44 genes with tumor-specific hypermethylation and gene expression changes >2-fold were enriched for cellular differentiation, development and transcription factor activity (S3 Table).

### Differences of DNA Methylation between Mammary Carcinomas Induced by Pre- and Post-Pubertal Exposure

**Fig 3:**
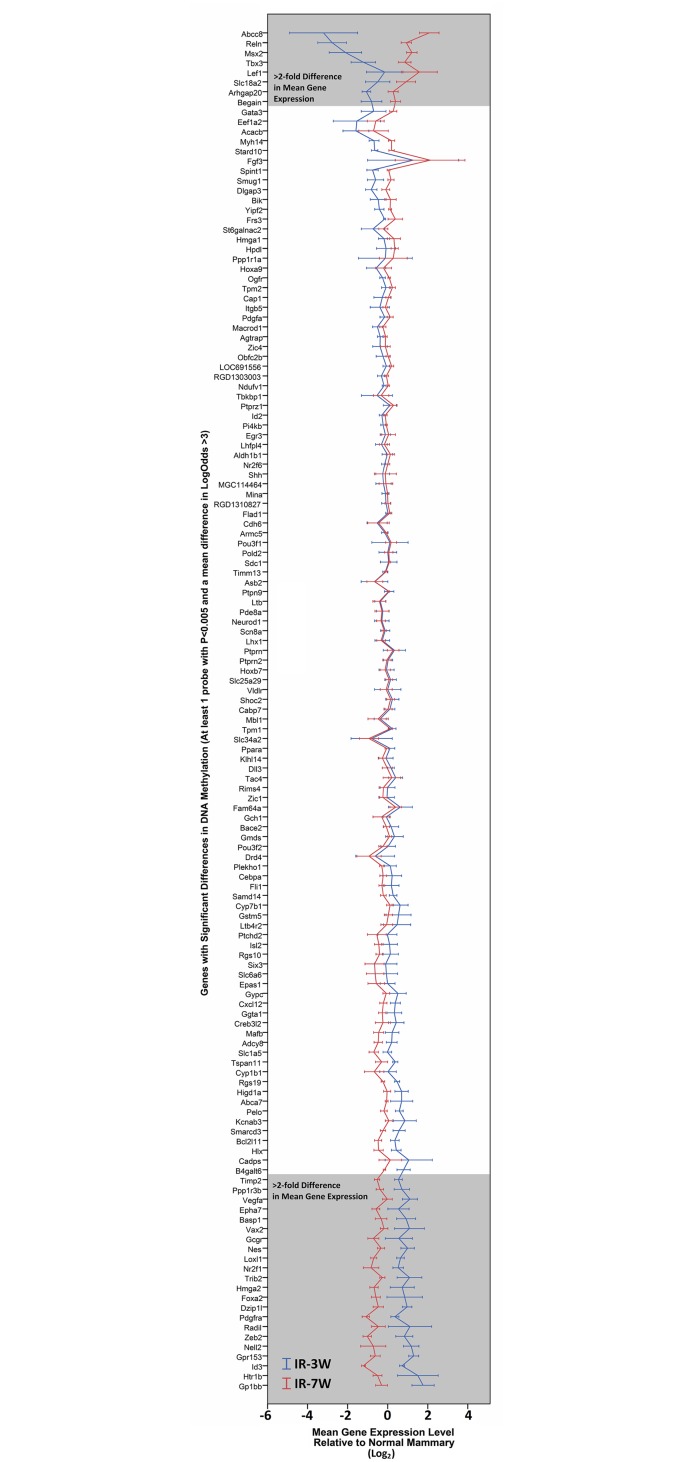
Gene expression levels by age at irradiation for candidate genes with age-specific DNA methylation. The mean gene expression levels (± standard error) in pre-pubertal (IR-3W, *n* = 4) and post-pubertal (IR-7W, *n* = 6) tumors are shown for the 223 genes which were selected as candidates showing age-specific DNA methylation (*P* < 0.005, mean LogOdds difference >3). The 30 genes which showed >2-fold changes in gene expression are shown within the grey boxed areas at the top (increased expression in post-pubertal tumors) and bottom of the plot (decreased expression in post-pubertal tumors).

Next, specific differences in DNA methylation patterns between mammary carcinomas induced by pre- or post-pubertal exposure were examined. Compared to the very distinct DNA methylation profiles in all tumors compared to normal mammary tissue, the methylation profiles in tumors arising in the three groups (pre-pubertal, post-pubertal and spontaneous) were very similar, with mean differences in methylation scores across all probes centered on zero (data not shown). However, there was still a group of genes which showed large, statistically significant changes (*P* < 0.005) between the two age-at-exposure groups (*n* = 223, Fig 3), and which had previously shown >2-fold differences in mean gene expression (*n* = 30). The LogOdds for CpG islands using the DNA methylation array were shown to be correlated with the DNA methylation level as assessed by bisulfite-sequencing using 4 different genes spanning a wide-range of methylation levels; and, comparison of the CpG island methylation levels measured here and the previously published gene expression levels showed that the candidate methylation-regulated genes exhibited different strengths and direction of the correlation consistent with the variable role of methylation in overall transcriptional control (S1 Fig).

However, these genes represented only 2% of the all of the genes which had >2-fold differences in mean gene expression (30/1593) between pre- and post-pubertal tumors. Parallel analysis excluding the single tumor with marked hypermethylation across the genome confirmed that the statistical significance threshold used was already sufficient to exclude changes which might have been due to large changes in this single sample.

Given that the balance of hormone receptor status of the tumors was known to be different between the two age-at-exposure groups, a univariate ANOVA including both PR status and age-at-exposure was conducted on these candidate genes to identify whether the DNA methylation differences were associated only with the irradiation age, only with the PR status, or both. Since none of the tumors arising after irradiation at 7 weeks of age were ER negative, it was not possible to separate the effects of age and ER status. All significant probes for the 30 candidate genes were significant only with age, except for *Epha7* (1/4 significant probes), *Timp2* (4/6 significant probes), and *Reln* (1/1 significant probe) which had probes that were significant for both age and PR status. None of the candidate probes showed a significant interaction between age and PR.

**Fig 4:**
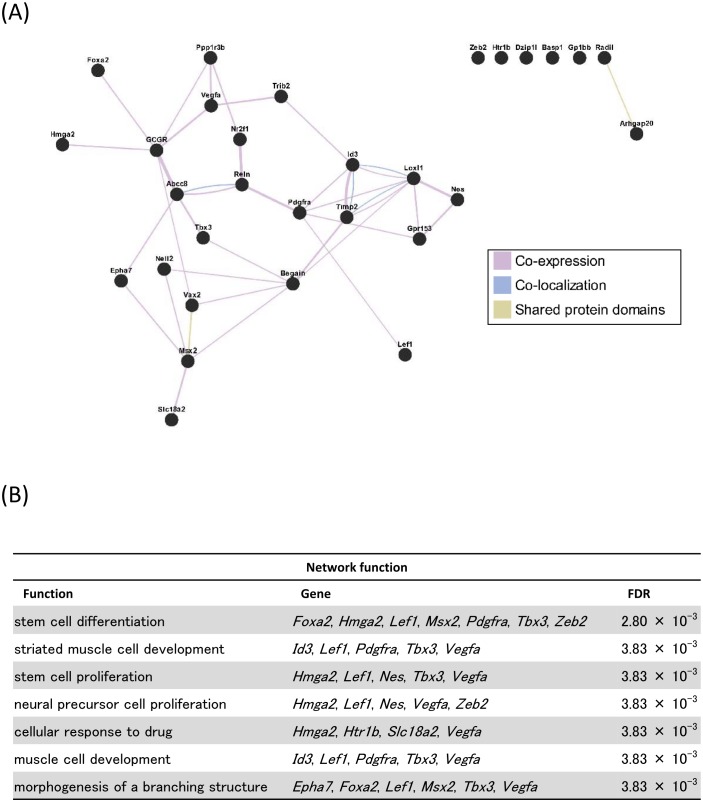
Functional analysis for genes with differential expression and methylation between mammary carcinomas induced by pre- and post-pubertal irradiation. (A) Interaction network between the 30 genes that were differentially expressed and methylated depending on age at exposure, with connections based on co-expression, co-localization or shared protein domains with increasing line thickness for stronger relationships. (B) Biological functions significantly enriched (False Discovery Rate, FDR < 0.05) within the 30 candidate genes, with the constituent genes listed. Most functional annotations rely on a sub-set of 7–8 common genes all within the stem cell differentiation class. Additional redundant and nested functions have been omitted for clarity.

Functional analysis revealed that the 30 candidate genes were enriched for stem cell differentiation roles, with seven of the genes in this category (*Foxa2*, *Hmga2*, *Lef1*, *Msx2*, *Pdgfra*, *Tbx3*, *Zeb2*) plus *Vegfa* also invoking additional gene ontology terms such as stem cell proliferation, branching morphogenesis, transcription factor regulation and muscle development (Fig 4 and S3 Table). The remaining genes were not enriched in any functional categories.

## Discussion

Largely, the mammary carcinomas showed global hypomethylation of genomic regions with low methylation in normal mammary tissue, a finding similar to that seen in human breast cancer, which has been shown to correlate with chromatin regions defined by histone modifications. The global DNA hypomethylation was observed across all of the spontaneous and radiation-induced carcinomas; however, there was a set of 44 genes which showed highly significant tumor-specific increases in DNA methylation (with changes in gene expression) that were enriched for cellular differentiation, development and transcription factor activity. This is consistent with the idea of global demethylation coupled with specific hypermethylation of genes which are important in establishing or maintaining tumor growth [33, 34].

Our selection criteria for age-specific methylation was chosen to limit the list to those genes with clear and large differences in the methylation levels for at least one CpG island probe, where we might expect the CpG island to have switched from methylated-to-unmethylated or vice versa [35]. At this level, 13.5% of the methylation candidates (30/223) also showed more than 2-fold differences in gene expression. Relaxing the methylation criteria increased the number of differentially-methylated candidate genes, but decreased the proportion of candidates with corresponding differences in gene expression, suggesting that our threshold was able isolate methylation differences with functional impact at the gene expression level.

Our hypothesis was that the set of genes which were differentially expressed between the tumors arising after pre- or post-pubertal irradiation might be the result of differing DNA methylation that might have existing in the cell of origin prior to tumor initiation, or that might have arisen in the changing microenvironment during puberty. The fact that only 2% of the genes with gene expression differences between the age groups were in our list of differentially-methylated genes suggests that DNA methylation is not a good explanation for the bulk of the observed differences. At the most relaxed DNA methylation criteria for the difference in the mean methylation levels between the 3 week and 7 week groups (*P* < 0.05), the expanded candidate list would still only have covered less than 10% of the >2-fold gene expression differences. Together, these data suggest that other regulatory mechanisms such as microRNA, chromatin and histone changes, transcription factor binding etc. might underlie the differences in the tumor phenotypes. So far, no obvious candidate signaling or transcription factors have been identified based on the relationships between the differentially expressed genes.

One of the difficulties in the analysis of both the gene expression and the methylation data is the different balance of hormone receptor status between the groups. Our multivariate analysis did not suggest that PR expression was responsible for or interacting with the DNA methylation levels of the genes in our final candidate list. However, we were not able to perform a similar analysis on other markers that might define various tumor subtypes, such as ER status or the potential cell of origin based on gene expression signature analysis. In order to establish these candidate DNA methylation differences as *bona fide* age-at-exposure depend changes will require analysis of larger sets of tumors where the various co-variate factors can be assessed in parallel.

The 30 genes that were in the final list of candidates consisted of 8 genes which were classified within multiple, overlapping gene ontology terms which were significantly enriched (stem cell differentiation/proliferation; morphogenesis of epithelial tubes/branching structures) while the remaining majority of the genes were not part of enriched gene ontology categories. It is possible that the differentially-methylated genes are related in a non-functional sense, i.e. they may share common genomic position or association with chromatin marks; however, even with our strict criteria the list may include false-positive changes that do not represent genuine age-related differences. One possible example is *Htr1b* which has been identified in previous breast cancer gene expression studies, but later shown to be a consequence of variable expression from the stromal population within a tumor sample [36]. Alternatively, these may be genuine DNA methylation differences between tumors induced by irradiation at different ages, but they may be secondary to another tumor trait that differs between the groups such as their tumor stage or metastatic potential. Future validation of these genes in independent sets of tumors, and in other mammary carcinoma models (mouse or human) would lend weight to their association with age at exposure. The *Foxa2*, *Msx2*, and *Zeb2* transcription factors are known to play a role in the epithelial-to-mesenchymal transition [37, 38] and *Reln* is known to be epigenetically regulated and correlates with prognostic factors in human breast cancer [39]. In addition, *Id3* and *Timp2* are associated with breast cancer metastasis [40, 41] and mammary gland differentiation [42, 43]. Furthermore, it has been reported that some of these genes (e.g. *Foxa2* and *Msx2*) are able to change gene expression patterns in human cancer [44, 45]. Thus, the genes with differential methylation-regulated gene expression include genes with important and relevant roles for cancer development and which might contribute to changes in gene expression of other genes that are not controlled by DNA methylation. However, the significance of this subset of genes and their secondary effects on the larger age-at-exposure gene signature are still unclear. Here, we have shown that rat mammary carcinomas induced by radiation exposure show the global DNA hypomethylation that is a common feature of human cancers including breast cancer, and that a set of specifically hypermethylated genes can also be identified which may point to their importance in mammary cancer development. Yet, the results also suggest that DNA methylation is not a likely mechanism underlying the differences in gene expression previously observed between tumors arising after pre- or post-pubertal irradiation, although a small number of candidates related to differentiation and morphogenesis may be worth further investigation to identify their role. Underlying molecular differences in mammary carcinomas induced by irradiation at different ages may help to reveal the mechanisms which mediate age-related radiation-induced breast cancer risk. However, the heterogeneity of human breast cancer means that the interpretation of molecular and phenotypic differences should be cautious, and take into account the co-variates that may influence the association with any one factor.

## Supporting Information

S1 Fig Comparison of DNA Methylation Array Data across Platforms. (DOCX)

S2 Fig Gene expression levels compared to normal mammary glands for candidate genes with tumor-specific DNA methylation. The mean gene expression levels (± standard error) in normal mammary gland (*n* = 3) and radiation-induced (*n* = 10) tumors are shown for the genes which were selected as candidates showing significant differential DNA methylation (*P* < 0.001, mean LogOdds difference >6). The 44 genes which showed >2-fold changes in gene expression are shown within the grey boxed areas at the top (increased expression in radiation-induced tumors) and bottom of the plot (decreased expression in radiation-induced tumors).
(TIF)

S1 Table Detailed information of normal tissues and mammary tumors analyzed in the present study. (XLS)

S2 Table Primer sets for bisulfite sequencing. (XLS)

S3 Table List of genes used by GeneMANIA and its molecular functions. (XLS)
