# Supplementary material for: DNA Methylation Patterns in Rat Mammary Carcinomas Induced by Pre- and Post-Pubertal Irradiation
Source: PLoS One. 2016 Oct 6;11(10):e0164194. doi: 10.1371/journal.pone.0164194 (PMC5053445; doi:10.1371/journal.pone.0164194)
Supplement: S1 Fig — (DOCX) [file pone.0164194.s001.docx]

**Methylation: Array vs Bisulfite Sequencing**

**(B)**

**Global Methylation: Array vs LINE1**

**(A)**


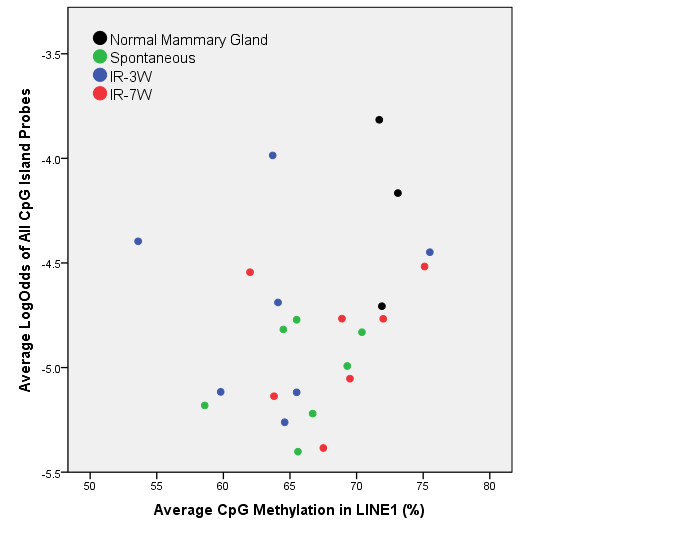

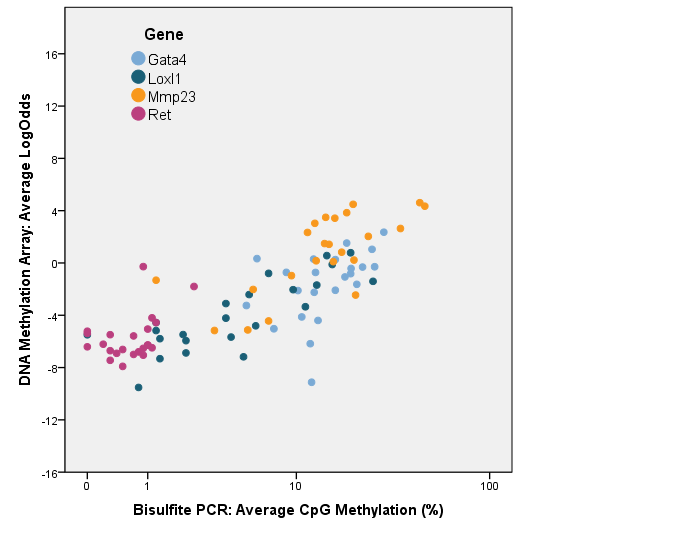


(B) To validate the analysed data from the DNA methylation array, the CpG islands from four genes were also assessed by bisulfite-sequencing. The data show that individually and collectively, the LogOdds closely reflects the degree of CpG island methylation.

(A) The LINE1 repeat element is frequently used as a surrogate for global methylation as it is present in thousands of copies throughout the genome. Plotting the average LogOdds for all CpG island probes (as a point-measurement of global methylation) against the average CpG methylation in LINE1 (18 CpG in a 387 bp PCR fragment) using bisulfite-sequencing shows that while an association is present and the demethylation in tumors can be seen with both measures, that much information is lost when compressing genome-wide methylation patterns into a single summary value.

**(C)**

**2**

**Correlations between DNA Methylation vs Previously Published Gene Expression Data**


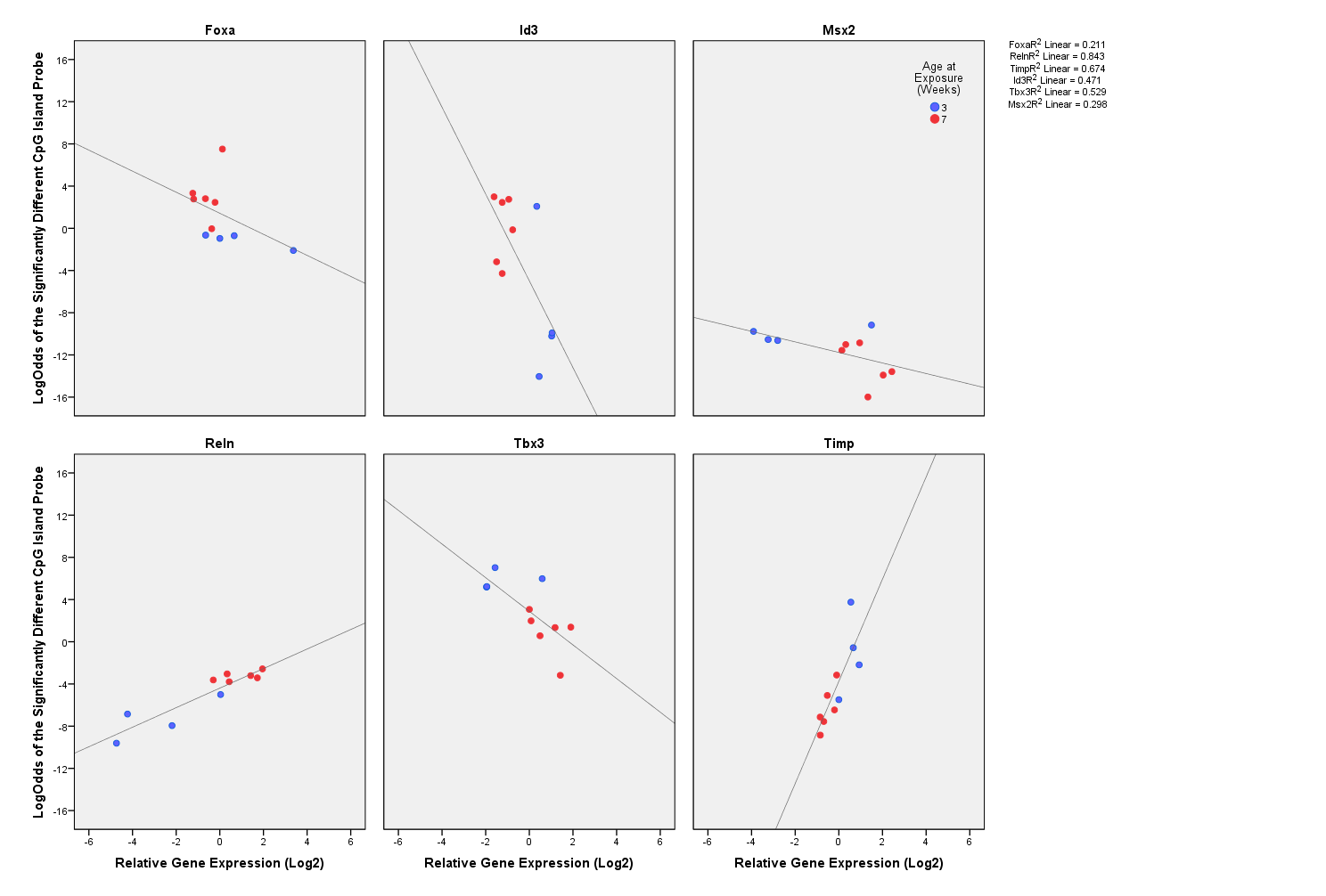


**2**

**R^2^ = 0.47**

**R^2^ = 0.67**

**R^2^ = 0.53**

**R^2^ = 0.84**

**R^2^ = 0.21**

**R^2^ = 0.30**

(C) To demonstrate the various relationships between the CpG island methylation and the levels of gene expression, the correlation between the methylation array and the previously published gene expression array data is shown for six of the candidate genes with differences with age at exposure. The lines show linear regressions between the LogOdds and the Log_2_ gene expression to highlight the varying strength and directions of the relationships (R^2^ values for each are shown), which likely depend on the regulatory mechanisms of each gene. Increased DNA methylation of CpG islands can result in increased gene expression (e.g. by preventing the expression of competing transcripts, preventing the binding of repressors).
