# Supplementary figures and images for: DNA Methylation Patterns in Rat Mammary Carcinomas Induced by Pre- and Post-Pubertal Irradiation
Source: PLoS One. 2016 Oct 6;11(10):e0164194. doi: 10.1371/journal.pone.0164194 (PMC5053445; doi:10.1371/journal.pone.0164194)

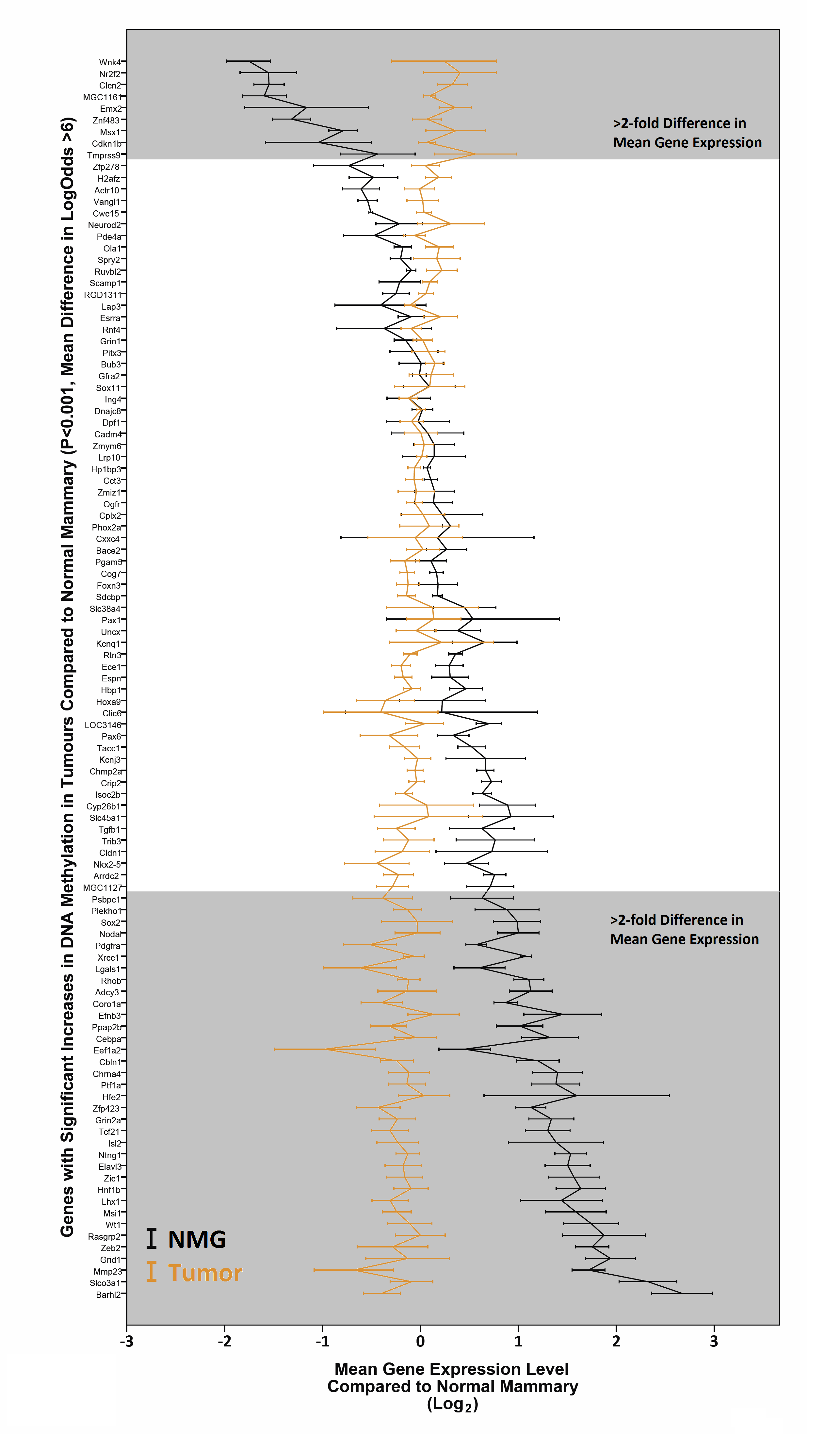

Supplement: S2 Fig — The mean gene expression levels (± standard error) in normal mammary gland (n = 3) and radiation-induced (n = 10) tumors are shown for the genes which were selected as candidates showing significant differential DNA methylation (P < 0.001, mean LogOdds difference >6). The 44 genes which showed >2-fold changes in gene expression are shown within the grey boxed areas at the top (increased expression in radiation-induced tumors) and bottom of the plot (decreased expression in radiation-induced tumors). (TIF) [file pone.0164194.s002.tif]
